# Appraisals of Social Trauma and Their Role in the Development of Post-Traumatic Stress Disorder and Social Anxiety Disorder

**DOI:** 10.3390/bs13070577

**Published:** 2023-07-11

**Authors:** Johann P. Hardarson, Berglind Gudmundsdottir, Audur G. Valdimarsdottir, Karen Gudmundsdottir, Arnrun Tryggvadottir, Kristjana Thorarinsdottir, Inga Wessman, Soley Davidsdottir, Gunnar Tomasson, Emily A. Holmes, Audur S. Thorisdottir, Andri S. Bjornsson

**Affiliations:** 1 Department of Psychology, University of Iceland, 102 Reykjavík, Iceland; 2 Department of Medicine, University of Iceland, 102 Reykjavík, Iceland; 3 Department of Mental Health Services, Landspitali—The National University Hospital of Iceland, 101 Reykjavík, Iceland; 4 Icelandic Center for Treatment of Anxiety Disorders, 108 Reykjavík, Iceland; 5 Department of Psychology, Uppsala University, 752 36 Uppsala, Sweden; 6 Department of Psychology, University of Regina, Regina, SK S4S 0A2, Canada

**Keywords:** appraisal, social trauma, social anxiety disorder, post-traumatic stress disorder

## Abstract

Cognitive theories of post-traumatic stress disorder (PTSD) feature appraisal of trauma as a critical factor in the development and maintenance of the disorder. Here we explored appraisals of social trauma (severe rejection or humiliation). Participants were outpatients with social anxiety disorder (SAD) and clinically significant PTSD symptoms (PTSS) after social trauma (n = 15); two clinical control groups of either SAD (n = 32) or obsessive-compulsive disorder (OCD; n = 13); and a control group with no diagnoses (n = 38). Measures included a clinical interview to assess social trauma and related open-ended appraisals and the Posttraumatic Cognitions Inventory (PTCI). Raters blind to group assignment performed content analyses of appraisals. Results showed that the PTSS group scored significantly higher than either clinical group on the PTCI SELF subscale. Only the SELF subscale predicted a diagnosis of both PTSS and SAD. All but one PTSS participant reported primarily negative beliefs about their social trauma, and the most common categories were flawed self and others are critical or cruel. Post-traumatic appraisals implicated in the course of PTSD are significant in how individuals respond to social trauma, with negative self-cognitions linked to both PTSS and SAD.

## 1. Introduction

### 1.1. Appraisal of Trauma

How an individual appraises a traumatic experience is critical in determining whether post-traumatic stress disorder (PTSD) develops. Ehlers and Clark [1] hypothesized that PTSD develops if the individual processes trauma in a way that evokes a sense of continuing threat. This perceived threat is a consequence of negative appraisals of the trauma, which can either be external (e.g., viewing the world or others as dangerous) or internal (e.g., considering oneself incapable). Consequently, the individual responds with maladaptive coping strategies, such as avoidance, which maintains PTSD. Cognitive models emphasize these appraisal processes in the onset and maintenance of PTSD [1,2,3,4], and this central thesis is well-supported in the trauma literature [5,6,7].

The Posttraumatic Cognitions Inventory (PTCI) was developed to assess appraisal processes post-trauma in PTSD [4]. They found three types of cognitions that contribute to the onset and maintenance of PTSD: Negative cognitions about the self (e.g., “I am inadequate”), the world (e.g., “The world is a dangerous place”), and self-blame (e.g., “The event happened because of the way I acted”). The PTCI discriminates well between traumatized individuals with PTSD and those without PTSD, and the cognitions assessed by the measure appear specific for PTSD [4]. The SELF subscale especially seems to have the strongest association with PTSD symptom severity [8,9]. To summarize, cognitive models suggest that what determines whether PTSD develops is the threat experienced during the event, in addition to dysfunctional appraisals of the event and, in particular, negative beliefs about the self, others, and the world.

### 1.2. Trauma and Threat Appraisal

The diagnostic criteria for PTSD in the *Diagnostic and Statistical Manual of Mental Disorders 5th edition (DSM-V)* specify that a person must have experienced trauma, defined as “exposure to actual or threatened death, serious injury, or sexual violence” [10]. Some have argued that this definition of trauma is too narrow and that other types of stressful experiences can lead to symptoms of PTSD [11,12], resulting in a debate often referred to as the Criterion A debate [13,14].

Recently, we argued that a more constructive approach would be to conceptualize trauma in terms of perceived threat linked to reproductive success in the evolutionary history of our species [15]. This approach is more in line with current theoretical models of PTSD that emphasize the individual’s appraisal of the threat rather than the event itself [1]. Indeed, perceived life threat is one of the most consistent predictors of PTSD [16,17], but other types of threat likely play a role.

A few studies suggest that negative social experiences (e.g., bullying) can be traumatic. A study in a sample diagnosed with social anxiety disorder (SAD) found that one-third of participants that reported such experiences met the criteria for PTSD, except for Criterion A [18]. Another study with a community sample found that participants reporting negative social events scored higher on measures of SAD and PTSD symptoms than those reporting only Criterion A events [19]. Additionally, one-third of participants reported an adverse social event as the most distressing event they had experienced despite most also reporting Criterion A events. Socially threatening experiences (e.g., peer victimization, teasing, mental abuse, and public humiliation) are also related to the onset and development of SAD [20,21,22]. We also know that social threat descriptions are prevalent in the hotspots and intrusive memories of PTSD patients [23,24].

These results suggest that a threat to life is not the only type of threat that can cause PTSD symptoms. There could also be a social threat [15,25,26] characterized by rejection and humiliation. From an evolutionary standpoint, being excluded from one’s group can be highly threatening and related to the likelihood of reproductive success [27,28].

### 1.3. Cognitive Models of SAD and PTSD

Cognitive models of SAD have many elements in common with cognitive models of PTSD. As with trauma and PTSD, negative social experiences do not automatically lead to the development of SAD. In other words, it is not just the experience that increases the risk of people developing the disorder but how they react or appraise it [29,30,31,32]. Cognitive models posit that core beliefs about their self being inherently flawed and others being critical play a vital role in maintaining the disorder and that these fundamental assumptions often develop from early experiences in childhood [32,33]. There is an overlap in these core beliefs and assumptions in PTSD and SAD. However, to our knowledge, there is scant research on this bridge between the two disorders. Furthermore, it is important to determine if the fear in PTSD is more specifically related to the fear of physical attacks from others, compared to humiliation or rejection (as in SAD), and if there are any variations in the views about the self in PTSD and SAD.

### 1.4. Social Threat and Social Trauma

We recently explored social trauma (events involving severe rejection or humiliation and therefore high social threat) and its potential to cause post-traumatic stress symptoms (PTSS) among outpatients with a primary diagnosis of SAD compared to a clinical control group of individuals with obsessive-compulsive disorder (OCD) and a control group of individuals with no psychiatric disorders [15]. The results showed that participants from all three groups had experienced social trauma: 82% of the SAD group, 79% of the OCD clinical control group, and 63% of the control group. However, only participants in the SAD group reported significant PTSS, with a third meeting the criteria for PTSD or suffering from clinically significant PTSS, similar to previous findings on social trauma [18]. This relationship between social trauma, SAD, and PTSS led us to hypothesize that some individuals might suffer from one integrated condition rather than two separate disorders. In other words, some individuals react to social threats with a fear of other social situations, intrusive memories of the social trauma, avoidance of similar situations, and being highly vigilant of social threat. In the current paper, we aim to shed further light on the concept of social trauma by exploring post-traumatic appraisals in the same data set.

### 1.5. Aims of the Current Study

The purpose of the current study is to explore post-traumatic appraisals after experiencing social trauma. We aimed to explore whether the types of appraisals that have been identified in people with PTSD after Criterion A trauma (as measured by the PTCI) are also present in those with PTSS (aim 1) and SAD (aim 2) after social trauma. We compared PTCI scores in four groups reporting social trauma, identified based on DSM-IV criteria: individuals with SAD and PTSS (PTSS group), a clinical control group of individuals with SAD, a clinical control group of individuals with OCD, and a control group of individuals with no psychiatric disorders. The selection of the OCD group aimed to investigate whether social trauma is specifically associated with PTSS and SAD rather than being linked to psychopathology in a broader sense. Furthermore, we compared and categorized answers to open-ended appraisal questions in the four groups to capture alternative post-traumatic appraisals (aim 3).

## 2. Materials and Methods

### 2.1. Participants

The sample consisted of 98 participants who all reported social trauma. They were divided into four groups (see Table 1): the PTSS group (15 participants with SAD and either full criteria of PTSD or clinically significant PTSS in response to their most severe social trauma), the SAD clinical control group (32 participants with SAD as a primary diagnosis and no PTSS), the OCD clinical control group (13 participants with OCD as a primary diagnosis and no PTSS) and the control group (38 participants with no psychiatric diagnoses; see previous paper for a description of the different types of social trauma [15]). Participants in the PTSS and SAD groups were either in or seeking treatment at the Icelandic Center for Treatment of Anxiety Disorders (*Kvíðameðferðarstöðin; KMS*). In the OCD and control groups, participants were recruited using social media advertisements and bulletin boards. Clinical interviews took place at the psychology department of the University of Iceland or KMS. Data collection began in late 2015 and was completed in late 2017.

The same pool of participants in the SAD group, as reported in an earlier paper [15], has here been split into two groups depending on the presence of PTSS. The inclusion criterion for the PTSS group was meeting the criteria for PTSD on the DSM-IV (except for Criterion A) or having clinically significant PTSS in response to social trauma. Participants diagnosed with PTSD in response to a threat to life trauma (Criterion A) were excluded from the analysis (n = 4). Clinically significant PTSS were defined as meeting Criteria B1 (intrusive memories), at minimum two symptoms of avoidance of stimuli and numbing (Criterion C), and at least two symptoms of increased arousal (Criterion D), with a duration of more than one month (Criterion E) and symptoms causing significant distress and/or impairment (Criterion F; [34]). The inclusion criterion for the original SAD group was a primary diagnosis of SAD, and a diagnosis of OCD was an exclusion criterion. A primary diagnosis of OCD was the inclusion criterion for the original OCD clinical control group, and a diagnosis of SAD was an exclusion criterion. The inclusion criterion for the control group was no current psychiatric diagnoses.

All participants signed an informed consent sheet and received a 5000 ISK gift certificate. The National Bioethics Committee of Iceland (Number: VSNb2011010021/03.07) approved the study.

### 2.2. Measures

The Imagery and Social Trauma Interview is a semi-structured interview used to assess social trauma and related appraisals. The measure was based on other versions of imagery interviews and translated by the last author [21,35]. It was adapted to focus more specifically on social trauma. First, the interviewer asks the participant if they have ever been humiliated or rejected by other people during their lifetime. If endorsed, the participant is asked to describe the worst social trauma. After assessing the trauma, the interviewer asks how the experience is currently appraised (“what was the meaning of the experience?”) and what the participant believes was the worst part of the socially traumatic experience. If participants had difficulty comprehending the questions, they were asked follow-up questions such as “do you feel the experience says something about you, other people, the world, or the future”?

The Mini-International Neuropsychiatric Interview (MINI) is a structured diagnostic interview for Axis I psychiatric disorders according to the DSM-IV. It was used to characterize the clinical samples and to assess for inclusion and exclusion criteria (e.g., SAD, OCD, and PTSS). The MINI was slightly adapted in this study. To assess PTSS after social trauma, the PTSD section of the MINI was administered to all participants twice for Criterion A trauma and social trauma, respectively. MINI has been found to have strong reliability and validity in relation to the structured clinical interview for the DSM-IV [36]. The Icelandic version of the MINI has good convergent validity with self-report measures of anxiety and depression [37]. The inter-rater reliability in the MINI was high in this study, with the agreement between raters ranging from 91–100% in the original SAD group and 100% in the control group.

The Liebowitz Social Anxiety Scale (LSAS) is a 24-item semi-structured clinical interview that assesses anxiety in (0 = none to 3 = severe) and avoidance of (0 = none to 3 = usually) social situations in the past week on a four-point Likert scale [38]. Scores on the LSAS range from 0 to 144, with higher scores indicating more severe social anxiety symptoms. The scale is sensitive to change and has excellent internal consistency on different subscales (Cronbach’s alpha = 0.81–0.92 [39]). The Icelandic version had excellent internal consistency for all groups (α = 0.90–0.91). Inter-rater reliability for the Icelandic version was high on both subscales (i.e., the intraclass correlation coefficient [ICC] = 1.00 and 0.90 for anxiety and ICC = 0.91 and 0.94 for avoidance) and for the total score (i.e., the ICC was 0.98 and 0.92) for the control and SAD group, respectively. The LSAS total score was used here to assess the severity of social anxiety symptoms.

The Posttraumatic Cognitions Inventory (PTCI) is a 33-item self-report questionnaire that assesses trauma-related thoughts and beliefs [4]. Participants completed the measure keeping in mind their worst social trauma. Each item on the PTCI is rated on a seven-point Likert scale ranging from one (i.e., totally disagree) to seven (i.e., totally agree). Higher scores on the scale indicate stronger endorsement of negative cognitions. The measure has three factors: negative cognitions about self (SELF), negative cognitions about the world (WORLD), and self-blame. The psychometric properties of the PTCI appear to be adequate. In one study [4], Cronbach’s alpha for the three PTCI subscales and the total score ranged from 0.86 (Self-blame) to 0.97 (SELF). The PTCI also discriminates well between traumatized individuals with and without a diagnosis of PTSD. In this study, Cronbach’s α for the complete sample for the three PTCI subscales were: SELF, α = 96; WORLD, α = 94; and Self-blame, α = 0.79; and Total PTCI score (33 items), α = 0.97. Correlations between the subscales were: SELF and WORLD *r* = 0.72; SELF and Self-blame *r* = 0.68; WORLD and Self-blame *r* = 0.50 (*ps* > 0.001).

The Patient Health Questionnaire-9 (PHQ-9) is a 9-item self-report measure of depression symptoms and their severity with good internal reliability [40]. Items are rated on a four-point Likert scale from 0 (i.e., not at all) to 3 (i.e., nearly every day). Scores range from 0 to 27, with higher scores indicating greater severity of depressive symptoms. The Icelandic version of the PHQ-9 had good internal consistency in the original SAD group (α = 0.87) and the OCD group (α = 0.85) but was fair in the control group (α = 0.66).

The Quality of Life Scale (QOLS) is a 16-item self-report measure of life quality rated in the following domains: Social and communal activities, material and physical wellbeing, relationships, personal development and fulfillment, and recreation. Items are rated on a seven-point Likert scale from 1 (terrible) to 7 (delighted), and total scores range from 16–112, with a higher score suggesting more life satisfaction. The QOLS has good reliability and validity [41]. The Icelandic version used in the current study had good internal consistency in the original OCD and control group (α = 0.86–87) but fair in the original SAD group (α = 0.76).

The Sheehan Disability Scale (SDS) is a three-item self-report measure of functional impairment resulting from emotional symptoms in work/school, social-, and family life. The impairment is rated on an 11-point Likert scale from 0 (not at all) to 10 (extremely), and total scores range from 0 to 30. The SDS has shown good construct validity and high internal reliability [42].

### 2.3. Procedure

The interviews, namely the Imagery and Social Trauma Interview, MINI, and LSAS were conducted by experienced psychologists or advanced graduate students in clinical psychology in a single session lasting approximately two to three hours. They received weekly supervision on differential diagnoses and training from the last author (a licensed clinical psychologist), which included sitting in on an assessment session, listening to recordings of sessions, reviewing administration manuals, and mock interviews. Every assessment was documented on a computer using the RedCap database, an encrypted software, and stored on secure servers [43]. Assessors monitored participant distress after each section and offered breaks accordingly. All participants were offered an interview with an independent clinical psychologist if the interview caused them distress.

Content analyses were conducted to identify the primary content of open-ended appraisals of social trauma. These analyses were managed by the last author and two advanced graduate students in clinical psychology who were blind to group assignments when conducting the content analyses. Following a methodology described by Joffe and Yardley [44], we created separate themes for the appraisal of social trauma before examining the data by reviewing categories drawn from theoretical models of how SAD and PTSD develop and are maintained [1,4,29,30,45]. The coders subsequently categorized the data, and some themes were modified when not appropriate for this data set. If coders disagreed on categorizing a specific appraisal, it was discussed further until a consensus was reached. If the appraisal did not fit into any category (e.g., due to inadequate information), it was rated as uncodeable.

### 2.4. Statistical Analyses

A one-way between-subjects ANOVA for the four groups was conducted to compare scores on the PTCI with posthoc comparisons using the Bonferroni correction (Aim 1). We used logistic regression analyses to determine if the three types of cognitions on the PTCI could predict PTSS (aim 1) or SAD (aim 2) diagnoses and multiple regression analysis to examine the relationship between the PTCI and social anxiety severity (LSAS) while adjusting for symptoms of depression according to the PHQ-9 (aim 2). Descriptive statistics were used to characterize the four groups in terms of appraisal themes (aim 3).

## 3. Results

All participants were Icelandic, and there were no significant differences in age, gender, student, and relationship status between the four groups (see Table 1). There were minor differences regarding educational status, with participants in the SAD clinical control group being less likely to have completed junior college or more compared to the control group (*p* < 0.05). Participants in all three clinical groups also met the criteria for some other psychiatric disorders according to the MINI, most commonly major depressive disorder and generalized anxiety disorder (Table 1). Within the PTSS group, more than half (*n* = 9; 60%) reported that their social trauma occurred before the onset of SAD.

We conducted a comparison of various clinical characteristics among the four groups using the Bonferroni correction. Our analysis revealed significant differences in the mean scores of LSAS (Social Anxiety Disorder symptom severity), PHQ-9 (depression symptom severity), and SDS (functional impairment) between the three clinical groups and the control group. The clinical groups exhibited higher scores on LSAS, PHQ-9, and SDS, indicating greater symptom severity and functional impairment, whereas they showed lower scores on QOLS (quality of life). These findings are detailed in Table 1, and the observed differences were statistically significant (*ps* < 0.05–0.001). Within the three clinical groups, the PTSS and the SAD groups scored higher on the LSAS and lower on the QOLS (*ps* < 0.001) compared to the OCD group. Additionally, the PTSS group had significantly higher scores on the PHQ-9 compared to the SAD and OCD groups (*ps* < 0.05–0.001) and higher scores on the SDS compared to the OCD group (*p* < 0.05).

### 3.1. Do the PTCI Subscales Predict PTSS in Response to Social Trauma?

The PTCI total score and scores on all three subscales differed significantly in the four groups (aim 1; see Table 2). Post hoc comparisons using the Bonferroni correction showed a significant difference in PTCI scores in total score and all three subscales between the three clinical groups and the control group (*ps* < 0.001). The PTSS group scored significantly higher on the SELF subscale compared to the clinical control groups (*ps* < 0.05) but the WORLD- (*ps* = 0.27–1.0) and SELF-BLAME- (*ps* = 1.0) subscales, along with the total score (*ps* = 0.15–88), did not differ significantly between the three clinical groups.

The three PTCI subscales were entered into a logistic regression analysis with PTSS vs. non-PTSS as the criterion (Aim 1; see Table 3). The omnibus test of the model coefficients was significant [χ^2^ (3) = 24.53, *p* < 0.001] such that 75/77 of the non-PTSS group and 5/15 of the PTSS group were correctly classified with a cut value of 0.5, indicating that the model is more accurate in classifying non-PTSS participants. Results for the three subscales showed that only the SELF subscale made a significant independent contribution (OR = 4.22, 95% CI [1.55, 11.33]).

### 3.2. Do the PTCI Subscales Predict SAD?

The three PTCI subscales were also entered into a logistic regression analysis with SAD vs. non-SAD as the criterion (Aim 2; see Table 3). The omnibus test of the model coefficients was significant [χ^2^ (3) = 57.73, *p* < 0.001], with 40/47 of the non-SAD group and 38/45 of the SAD group being correctly classified according to a cut value of 0.5. Results for the three subscales showed that only the SELF subscale made a significant independent contribution (OR = 9.50, 95% CI [3.51–25.37]). It should be noted that the PTCI did not (in a separate analysis) predict the diagnosis of OCD [χ^2^ (3) = 3.44, *p* = 0.33].

Since the association between the PTCI and severity of social anxiety is also of interest, a multiple regression analysis was conducted for scores on the LSAS total score and subscale scores on the PTCI while controlling for symptoms of depression with scores from the PHQ-9 for all participants (see Table 4). A significant regression equation was found (*F*(4, 89) = 31.63, *p* < 0.001, *R*^2^ = 0.60). The SELF subscale was again the only scale found to be significant. With an increase of one unit on the SELF scale, there is a predicted increase in the LSAS total score of more than 13 points.

### 3.3. Open-Ended Appraisals of Social Trauma: Exploratory Descriptive Analysis

Using content analysis on open-ended questions on appraisals of social trauma, 19 different appraisal categories were identified in the PTSS group, 37 in the SAD clinical control group, 17 in the OCD clinical control group, and 40 in the control group (see Table 5). In the PTSS group, all but one appraisal were negative (n = 18; 94.7%). Appraisals were also primarily negative in the SAD group (n = 31; 83.8%) and OCD clinical control groups (n = 13; 76.5%) but rarely so in the control group (n = 6; 15%), in which the appraisals were mostly positive (n = 21; 55.3%) or neutral (n = 13; 34.2%). The most common appraisal category identified in the three clinical groups was flawed self, especially in the PTSS group, where 73.3% endorsed such a view of self. The category others are critical and/or cruel was also more commonly reported in the PTSS group than in the SAD and OCD groups, and less commonly, that the world is dangerous.

## 4. Discussion

### 4.1. Post-Traumatic Appraisals in the Development of PTSS after Social Trauma

What kind of beliefs do individuals associate with socially traumatic experiences? We aimed to explore post-traumatic appraisals of social trauma (perceived rejection or humiliation) and their association with PTSS and SAD. We compared appraisals after social trauma in four groups: Individuals with SAD and clinically significant PTSD symptoms (PTSS group), individuals with SAD and no PTSS (SAD clinical control group), individuals with OCD and no PTSS (OCD clinical control group), and a control group with no disorders.

We found that the PTCI did discriminate between the PTSS and the control group, with the PTSS group scoring significantly higher on all three subscales (aim 1). In addition, mean scores on the subscales in the PTSS group were similar to those reported in other PTSD samples [8,46,47]. The SELF subscale was significantly higher in the PTSS group compared to the SAD- and OCD clinical control groups, which confirms the importance of a negative view of self in the development and maintenance of PTSD, which has been found repeatedly in the PTSD appraisal literature [8,9,46]. Surprisingly, scores on the PTCI for both clinical control groups were relatively high compared to other samples exposed to Criterion A trauma with no PTSS [4,8,46]. We found no statistical differences between the three clinical groups on the WORLD and Self-blame subscales. Additionally, the PTCI scale was more accurate in predicting non-PTSS than PTSS, which may relate to the small sample size in the PTSS group and high scores in the clinical control groups. Based on these results, the PTCI does not fully discriminate between socially traumatized individuals with and without PTSS, but more studies with larger samples are needed.

It is important to explore these and other appraisal processes more carefully. Our third aim was to conduct an exploratory analysis of possible nuances in post-traumatic appraisals of social trauma by asking open-ended questions about the primary meaning of the experience and conducting content analyses to categorize appraisals. The results showed that the PTSS group reported much more negative beliefs than the control group, which reported mainly neutral or positive beliefs in relation to their worst social trauma. All but one in the PTSS group reported negative appraisals compared to less than one-fifth in the control group. Most participants in the PTSS group (75%) said their worst social trauma made them adopt or strengthen beliefs that they were inherently flawed. Furthermore, 31% of participants in the PTSS group said that other people were generally critical or cruel. Surprisingly few (n = 2) in the PTSS group reported beliefs about the world being unsafe and dangerous, given that this is theorized to be a common belief in individuals diagnosed with PTSD [1,4]. This finding demonstrates the importance of investigating differences in post-traumatic appraisals after social trauma compared to threat-to-life trauma.

Negative self-appraisals are common in both PTSD and SAD, with theoretical models of these disorders highlighting the maladaptive role of cognitions, such as seeing oneself as flawed or incompetent in the course of both disorders [4,29,45,48]. Negative perceptions of the self are also commonplace in OCD (e.g., being immoral and unworthy [49]), although not as central in cognitive models of the disorder [50,51]. Viewing the world as physically unsafe, however, is generally more specific to PTSD [4,52], although considering others hostile is related to SAD [53] and general overestimation of threat to OCD [54], which could contribute to all three groups having high scores on the world subscale of the PTCI. Social trauma seems to induce similar levels of self-blame in clinical groups as Criterion A trauma, although direct comparisons are needed. The role of self-blame is unclear, given that the self-blame subscale did not predict symptoms of PTSS or SAD in this study. Other studies have found that this subscale does not correlate with increased PTSD symptoms [8,46].

We conclude that the belief that the self is inherently flawed and that others are critical or cruel is more likely associated with PTSS after social trauma than after a life-threatening experience, although more studies directly comparing these two types of threat are needed. The treatment implications might be that strategies targeting such appraisals could benefit individuals suffering from PTSS and SAD symptoms in response to social trauma. One recent low-intensity digitized tool that could be deployed is cognitive bias modification for appraisals (CBM-APP), in which appraisals are targeted directly [55] and could, in theory, be adapted further to appraisals associated with PTSS after social trauma.

### 4.2. PTSD and SAD

PTSD and SAD are highly comorbid disorders [56], and various attempts have been made to explain this relationship, e.g., genetic and psychological vulnerabilities [57]. Indeed, our results showed few significant differences in the PTCI and open-ended appraisal categories between SAD participants with and without PTSS. Additionally, post-traumatic appraisals, predominantly negative views of the self, as measured with the PTCI, were also strongly linked to symptoms of SAD (aim 2).

Our previous report found that only individuals with SAD as a primary diagnosis and not OCD (the clinical control group) developed PTSS in response to their most severe social trauma [15]. We hypothesized that, rather than suffering from two different disorders, some individuals might present with an integrated condition of SAD and PTSD in response to social trauma. Here, we found that negative beliefs about the self and of others being critical or cruel could be another bridge between these two disorders.

### 4.3. Study Limitations and Strengths

There are several limitations to the study. The sample sizes in the PTSS and OCD groups were small, affecting statistical power, meaning that results should be treated cautiously. Future studies should incorporate larger samples that would, for example, allow for analyses to control for depression symptoms. However, it should be noted that in the one analysis in which statistical power did allow for controlling for depression symptoms, the association between the SELF scale and the severity of social anxiety symptoms still held. We used the MINI for DSM-IV to assess for PTSS, the most recent diagnostic interview validated in Iceland. As there is overlap in symptom presentation in the clinical groups, future studies should include a PTSS group without SAD to investigate whether the impact of social trauma observed in this study extends more broadly. Future investigations should also use the DSM-5 and a more sensitive measure of PTSS, such as The Clinician-Administered PTSD Scale for DSM-5 (CAPS-5; [58]). The strengths of this study include using clinical interviews instead of relying solely on self-report questionnaires and the careful training and supervision of the assessment team.

## 5. Conclusions

The current study is the first to our knowledge to assess post-traumatic appraisals of social trauma. Negative cognitions about the self seem to be especially important in the development of PTSS in response to social trauma, particularly seeing oneself as flawed. As the PTCI did not fully discriminate between those with and without symptoms of PTSD, it is critical to research nuances in these appraisal processes. We found evidence that beliefs about the self being flawed and on others being critical and cruel may be a bridge between PTSD and SAD, such that many individuals react to social trauma by developing both PTSD and SAD symptoms as one integrated condition rather than two separate disorders. More research is needed on these important questions, which are likely to have important implications for both theoretical models and the treatment of individuals that report both PTSS and SAD symptoms in response to social trauma.

## Figures and Tables

**Table 1:** Clinical characteristics (N = 98).

	PTSS (n = 15)	SAD (n = 32)	OCD (n = 13)	Control (n = 38)	Chi-Square- or *F* Statistic
Demographic variables ^a^					
Age (*M*; *SD*)	29.8 (11.9)	30.6 (11.4)	29.7 (8.2)	30.3 (9.2)	*F* (3, 97) = 0.04
Gender (% female)	11 (73.3)	17 (53.1)	10 (76.9)	20 (52.6)	*Χ*^2^ (3, 98) = 4.1
Education (% Junior College or more)	8 (53.3)	14 (43.8)	8 (61.5)	31 (81.6)	*Χ*^2^ (3,98) = 11.2 **
Currently a student (%)	7 (50.0) ^c^	11 (35.5) ^c^	7 (53.8)	13 (35.1) ^c^	*Χ*^2^ (3, 95) = 2.3
Married or living with a partner (%)	6 (40.0)	19 (59.4)	8 (61.5)	24 (63.2)	*Χ*^2^ (3, 98) = 2.5
Psychiatric Comorbidity ^b^					
Major depressive disorder	7 (46.7)	10 (31.3)	3 (23.1)	-	-
Dysthymia	-	1 (3.1)	-		
Bipolar I disorder	-	1 (3.1)	-	-	-
Bipolar II disorder	-	2 (6.3)	2 (15.4)	-	-
Panic disorder with agoraphobia	1 (6.7)	-	3 (23.1)	-	-
Agoraphobia without panic	-	-	3 (23.1)	-	-
Social anxiety disorder	15 (100)	32 (100)	-	-	-
Obsessive compulsive disorder	-	-	13 (100)	-	-
Post-traumatic stress disorder (social trauma)	13 (86.7)	-	-	-	-
Alcohol dependence	1 (6.7)	2 (6.3)	1 (7.7)	-	-
Alcohol abuse	-	1 (3.1)	1 (7.7)	-	-
Drug dependence	-	2 (6.3)	-	-	-
Drug abuse	-	-	-	-	-
Bulimia	-	-	2 (15.4)	-	-
Anorexia nervosa	-	-	1 (7.7)		
Generalized anxiety disorder	2 (13.3)	3 (9.4)	4 (30.8)	-	-
Other clinical characteristics					
LSAS ^e^	85.1 (16.5)	79.6 (19.0)	45.4 (12.9) ^d^	12.8 (10.3)	*F* (3, 95) = 147.1 ***
PHQ-9 ^f^	15.3 (6.2) ^c^	8.8 (5.3) ^c^	9.3 (6.4) ^d^	1.7 (2.1)	*F* (3, 93) = 34.8 ***
QOLS ^g^	64.0 (12.7) ^c^	62.5 (11.8) ^c^	77.8 (15.5) ^d^	95.0 (8.7)	*F* (3, 93) = 55.5 ***
SDS ^h^	205.7 (41.9) ^c^	180.9 (58.6) ^c^	149.9 (77.8) ^d^	9.2 (22.2)	*F* (3, 93) = 100.8 ***

Note. ** *p* < 0.05 *** *p* < 0.001 ^a^ Results in the table are presented as n (%) or mean (standard deviation). ^b^ The Mini International Neuropsychiatric Interview was used to assess all disorders. ^c^ One missing value. ^d^ Two missing values. ^e^ LSAS = Liebowitz Social Anxiety Scale. ^f^ PHQ-9 = Patient Health Questionnaire-9. ^g^ QOLS = Quality of Life Scale. ^h^ SDS = Sheehan Disability Scale. PTSS = posttraumatic stress symptoms, SAD = social anxiety disorder, OCD = obsessive compulsive disorder.

**Table 2:** Trauma-related thoughts and beliefs of the study participants.

	PTSS (n = 15)	SAD (n = 30) ^d^	OCD (n = 11) ^d^	Controls (n = 36) ^d^	*F* Statistic
PTCI subscales	*M* (*SD*)	*M* (*SD*)	*M* (*SD*)	*M* (*S.D.*)	
Negative cognitions about self	4.00 (1.09) ^a^	3.28 (0.74) ^b^	2.89 (0.79) ^b^	1.53 (0.53) ^c^	*F*(3, 91) = 50.91 ***
Negative cognitions about the world	5.00 (1.07) ^a^	4.20 (1.22) ^a^	4.83 (1.49) ^a^	2.68 (1.28) ^b^	*F*(3, 91) = 17.60 ***
Self-blame	4.01 (1.19) ^a^	3.81 (1.34) ^a^	3.49 (1.51) ^a^	2.24 (0.83) ^b^	*F*(3, 91) = 13.60 ***
Total score	139.13 (31.12) ^a^	120.77 (28.32) ^a^	118.00 (35.64) ^a^	62.19 (20.50) ^b^	*F*(3, 91) = 41.52 ***

Note. Results are expressed as mean and standard deviations of the subscales of the PTCI instrument and the total score. PTCI = Posttraumatic Cognitions Inventory. PTSS = Posttraumatic Stress Symptoms, SAD = social anxiety disorder, OCD= obsessive compulsive disorder *M* = mean, *SD* = standard deviation. *** *p* < 0.001. Means with different subscripts (a, b, or c) are significantly different. ^d^ Two missing values.

**Table 3:** The association of trauma-related thoughts and beliefs with PTSS and SAD.

	PTSS		
Subscale of the PTCI	OR	Wald-Statistic	95% Confidence Interval for OR
Negative cognitions about self	4.22	7.96 *	1.55–11.33
Negative cognitions about the world	1.22	0.30	0.60–2.44
Self-blame	0.86	0.25	0.47–1.57
	SAD		
Negative cognitions about self	9.50	19.61 **	3.51–25.37
Negative cognitions about the world	0.67	2.31	0.40–1.12
Self-blame	1.07	0.06	0.62–1.86

Note. The associations are expressed as odds ratios of PTSS or SAD for each unit increase in the subscales of the PTCI instrument. PTCI = Posttraumatic Cognitions Inventory. PTSS = Posttraumatic Stress Symptoms. SAD = social anxiety disorder. OR = odds ratio. * *p* < 0.01 ** *p* > 0.001.

**Table 4:** The association of trauma-related thoughts and beliefs with symptoms of social anxiety.

Subscale of the PTCI	*β*	*S.E.*	*t*	95% Confidence Interval for *β*
Negative cognitions about self	13.29	3.69	3.60 **	5.95–20.63
Negative cognitions about the world	0.76	2.35	0.33	−3.91–5.43
Self-blame	1.95	2.42	0.80	−2.87–5.43
PHQ-9 total score	1.65	0.52	3.17 *	6.14–2.68

Note. Results are expressed as linear regression coefficients of the subscale scores of the PTCI instrument with the total score of the LSAS instrument. The analysis was adjusted for depressive symptoms. LSAS = The Liebowitz Social Anxiety Scale. PTCI = Posttraumatic Cognitions Inventory. *β* = slope, *SE* = standard error, *t* = *t*-test, * < 0.01, ** < 0.001.

**Table 5:** Appraisal categories of social trauma.

Appraisal Theme	PTSS (n = 15)	SAD (n = 32)	OCD (n = 13)	Control (n = 38)
Flawed self	11 (73.3%)	13 (40.6%)	7 (53.4%)	-
Others are critical and/or cruel	5 (33.3%)	5 (15.6%)	1 (7.7%)	2 (5.3%)
The world is dangerous	2 (13.3%)	7 (21.9%)	3 (23.1%)	4 (10.5%)
I am different from others	-	3 (9.4%)	1 (7.7%)	-
Self-blame	-	1 (3.1%)	1 (7.7%)	-
I am not capable	-	2 (6.3%)	-	-
Neutral or no meaning	-	6 (18.8%)	-	13 (34.2%)
Strong self	-	-	4 (30.7%)	19 (50.0%)
Optimism about the future	1 (6.7%)	-	-	-
Positive beliefs about other people	-	-	-	2 (5.3%)
Uncodeable	-	2 (6.3%)	-	-

Note. Some participants (PTSS group: n = 4; SAD group: n = 7; OCD group: n = 2; control group: n = 2) reported two primary appraisals of their socially traumatic event/experience; the percentages reveal the percentage of individuals who reported a given appraisal category within a group. PTSS = Posttraumatic Stress Symptoms, SAD = Social anxiety disorder, OCD = obsessive compulsive disorder.

## Data Availability

The data are not publicly available due to legal and privacy issues.

## References

[1] Ehlers A., Clark D.M. (2000). A cognitive model of posttraumatic stress disorder. Behav. Res. Ther..

[2] Beck J.G., Jabobs-Lentz J., McNiff J., Olsen S.A., Clapp J.D., Zoellner L., Feeny N.C. (2011). Understanding Post-Trauma Cognitions and Beliefs. Facilitating Resilience and Recovery Following Traumatic Events.

[3] Brewin C.R., Holmes E.A. (2003). Psychological theories of posttraumatic stress disorder. Clin. Psychol. Rev..

[4] Foa E.B., Ehlers A., Clark D.M., Tolin D.F., Orsillo S.M. (1999). The Posttraumatic Cognitions Inventory (PTCI): Development and validation. Psychol. Assess..

[5] Dunmore E., Clark D.M., Ehlers A. (1999). Cognitive factors involved in the onset and maintenance of posttraumatic stress disorder (PTSD) after physical or sexual assault. Behav. Res. Ther..

[6] Halligan S.L., Michael T., Clark D.M., Ehlers A. (2003). Posttraumatic stress disorder following assault: The role of cognitive processing, trauma memory, and appraisals. J. Consult. Clin. Psychol..

[7] Lancaster C.L., Cobb A.R., Lee H.-J., Telch M.J. (2016). The role of perceived threat in the emergence of PTSD and depression symptoms during warzone deployment. Psychol. Trauma Theory Res. Pract. Policy.

[8] Beck J.G., Coffey S.F., Palyo S.A., Gudmundsdottir B., Miller L.M., Colder C.R. (2004). Psychometric Properties of the Posttraumatic Cognitions Inventory (PTCI): A Replication With Motor Vehicle Accident Survivors. Psychol. Assess..

[9] Moser J.S., Hajcak G., Simons R.F., Foa E.B. (2007). Posttraumatic stress disorder symptoms in trauma-exposed college students: The role of trauma-related cognitions, gender, and negative affect. J. Anxiety Disord..

[10] American Psychiatric Association (2013). Diagnostic and Statistical Manual of Mental Disorders: DSM-5.

[11] Roberts A.L., Dohrenwend B.P., Aiello A.E., Wright R.J., Maercker A., Galea S., Koenen K.C. (2012). The Stressor Criterion for Posttraumatic Stress Disorder: Does It Matter?. J. Clin. Psychiatry.

[12] Van Hooff M., McFarlane A.C., Baur J., Abraham M., Barnes D.J. (2009). The stressor Criterion-A1 and PTSD: A matter of opinion?. J. Anxiety Disord..

[13] Boals A., Schuettler D. (2009). PTSD symptoms in response to traumatic and non-traumatic events: The role of respondent perception and A2 criterion. J. Anxiety Disord..

[14] Stein J.Y., Wilmot D.V., Solomon Z. (2016). Does one size fit all? Nosological, clinical, and scientific implications of variations in PTSD Criterion A. J. Anxiety Disord..

[15] Bjornsson A.S., Hardarson J.P., Valdimarsdottir A.G., Gudmundsdottir K., Tryggvadottir A., Thorarinsdottir K., Wessman I., Sigurjonsdottir Ó., Davidsdottir S., Thorisdottir A.S. (2020). Social trauma and its association with posttraumatic stress disorder and social anxiety disorder. J. Anxiety Disord..

[16] Larsen S.E., Berenbaum H. (2017). Did the DSM-5 Improve the Traumatic Stressor Criterion?: Association of DSM-IV and DSM-5 Criterion A with Posttraumatic Stress Disorder Symptoms. Psychopathology.

[17] Pinto R.J., Henriques S.P., Jongenelen I., Carvalho C., Maia C. (2015). The Strongest Correlates of PTSD for Firefighters: Number, Recency, Frequency, or Perceived Threat of Traumatic Events?: The Strongest Correlates of PTSD for Firefighters. J. Trauma. Stress.

[18] Erwin B.A., Heimberg R.G., Marx B.P., Franklin M.E. (2006). Traumatic and socially stressful life events among persons with social anxiety disorder. J. Anxiety Disord..

[19] Carleton R.N., Peluso D.L., Collimore K.C., Asmundson G.J. (2011). Social anxiety and posttraumatic stress symptoms: The impact of distressing social events. J. Anxiety Disord..

[20] Chartier M.J., Walker J.R., Stein M.B. (2001). Social phobia and potential childhood risk factors in a community sample. Psychol. Med..

[21] Hackmann A., Clark D., McManus F. (2000). Recurrent images and early memories in social phobia. Behav. Res. Ther..

[22] McCabe R.E., Miller J.L., Laugesen N., Antony M.M., Young L. (2010). The relationship between anxiety disorders in adults and recalled childhood teasing. J. Anxiety Disord..

[23] Grey N., Young K., Holmes E. (2002). Cognitive restructuring within reliving: A treatment for peritraumatic emotional “hotspots” in posttraumatic stress disorder. Behav. Cogn. Psychother..

[24] Holmes E.A., Grey N., Young K.A. (2005). Intrusive images and “hotspots” of trauma memories in Posttraumatic Stress Disorder: An exploratory investigation of emotions and cognitive themes. J. Behav. Ther. Exp. Psychiatry.

[25] Bjornsson A.S., Bjornsson A.S., Eydal G., Kristjansdottir K. (2016). Maðurinn er Félagslegt Dýr. Af sál.

[26] Neuner F. (2023). Physical and social trauma: Towards an integrative transdiagnostic perspective on psychological trauma that involves threats to status and belonging. Clin. Psychol. Rev..

[27] Gilbert P. (2002). Evolutionary Approaches to Psychopathology and Cognitive Therapy. J. Cogn. Psychother..

[28] Gilboa-Schechtman E., Shachar I., Helpman L., Hofmann S.G., DiBartolo P.M. (2014). Chapter 21—Evolutionary Perspective on Social Anxiety. Social Anxiety.

[29] Clark D.M., Wells A., Heimberg R.G., Liebowitz M.R., Hope D.A., Schneier F.R. (1995). A cognitive model of social phobia. Social Phobia: Diagnosis, Assessment, and Treatment.

[30] Heimberg R.G., Brozovich F.A., Rapee R.M., Hofmann S.G., DiBartolo P.M. (2012). A Cognitive Behavioral Model of Social Anxiety Disorder: Update and Extension. Social Anxiety: Clinical, Developmental, and Social Perspectives.

[31] Levinson C.A., Langer J.K., Rodebaugh T.L. (2013). Reactivity to Exclusion Prospectively Predicts Social Anxiety Symptoms in Young Adults. Behav. Ther..

[32] Spence S.H., Rapee R.M. (2016). The etiology of social anxiety disorder: An evidence-based model. Behav. Res. Ther..

[33] Calvete E., Orue I., Hankin B.L. (2013). Early maladaptive schemas and social anxiety in adolescents: The mediating role of anxious automatic thoughts. J. Anxiety Disord..

[34] Association A.P. (2000). Diagnostic and Statistical Manual of Mental Disorders.

[35] Lipton M.G., Brewin C.R., Linke S., Halperin J. (2010). Distinguishing features of intrusive images in obsessive–compulsive disorder. J. Anxiety Disord..

[36] Sheehan D., Lecrubier Y., Sheehan K.H., Janavs J., Weiller E., Keskiner A., Schinka J., Knapp E., Sheehan M., Dunbar G. (1997). The validity of the Mini International Neuropsychiatric Interview (MINI) according to the SCID-P and its reliability. Eur. Psychiatry.

[37] Sigurðsson B.H. (2008). Samanburður á tveimur stöðluðum greiningarviðtölum og tveimur sjálfsmatskvörðum: MINI, CIDI, PHQ og DASS.

[38] Liebowitz M.R. (2003). Guidelines for Using the Liebowitz Social Anxiety Scale. https://heilbrigdisvisindastofnun.hi.is/files/2022-12/LSAS_Manual.pdf.

[39] Heimberg R.G., Horner K.J., Juster H.R., Safren S.A., Brown E.J., Schneier F.R., Liebowitz M.R. (1999). Psychometric properties of the Liebowitz Social Anxiety Scale. Psychol. Med..

[40] Kroenke K., Spitzer R.L., Williams J.B. (2001). The PHQ-9: Validity of a brief depression severity measure. J. Gen. Intern. Med..

[41] Liedberg G.M., Burckhardt C.S., Henriksson C.M. (2005). Validity and reliability testing of the Quality of Life Scale, Swedish version in women with fibromyalgia—Statistical analyses. Scand. J. Caring Sci..

[42] Leon A.C., Olfson M., Portera L., Farber L., Sheehan D.V. (1997). Assessing Psychiatric Impairment in Primary Care with the Sheehan Disability Scale. Int. J. Psychiatry Med..

[43] Harris P.A., Taylor R., Thielke R., Payne J., Gonzalez N., Conde J.G. (2009). Research electronic data capture (REDCap)—A metadata-driven methodology and workflow process for providing translational research informatics support. J. Biomed. Inform..

[44] Joffe H., Yardley L., Marks D.F., Yardley L. (2004). Content and Thematic Analysis. Research Methods for Clinical and Health Psychology.

[45] Moscovitch D.A. (2009). What Is the Core Fear in Social Phobia? A New Model to Facilitate Individualized Case Conceptualization and Treatment. Cogn. Behav. Pract..

[46] Startup M., Makgekgenene L., Webster R. (2007). The role of self-blame for trauma as assessed by the Posttraumatic Cognitions Inventory (PTCI): A self-protective cognition?. Behav. Res. Ther..

[47] Karl A., Rabe S., Zöllner T., Maercker A., Stopa L. (2009). Negative self-appraisals in treatment-seeking survivors of motor vehicle accidents. J. Anxiety Disord..

[48] Janoff-Bulman R., Stamm B.H. (1996). Psychometric Review of the World Assumptions Scale. Measurement of Stress, Trauma, and Adaptation.

[49] Doron G., Kyrios M. (2005). Obsessive compulsive disorder: A review of possible specific internal representations within a broader cognitive theory. Clin. Psychol. Rev..

[50] Rachman S. (1998). A Cognitive Theory of Obsessions. Behavior and Cognitive Therapy Today.

[51] Salkovskis P.M. (1985). Obsessional-compulsive problems: A cognitive-behavioural analysis. Behav. Res. Ther..

[52] Owens G.P., Pike J.L., Chard K.M. (2001). Treatment effects of cognitive processing therapy on cognitive distortions of female child sexual abuse survivors. Behav. Ther..

[53] DeWall C.N., Buckner J.D., Lambert N.M., Cohen A.S., Fincham F.D. (2010). Bracing for the worst, but behaving the best: Social anxiety, hostility, and behavioral aggression. J. Anxiety Disord..

[54] Obsessive Compulsive Cognitions Working Group (1997). Cognitive assessment of obsessive-compulsive disorder. Behav. Res. Ther..

[55] Woud M.L., Blackwell S.E., Shkreli L., Würtz F., Cwik J.C., Margraf J., Holmes E.A., Steudte-Schmiedgen S., Herpertz S., Kessler H. (2021). The Effects of Modifying Dysfunctional Appraisals in Posttraumatic Stress Disorder Using a Form of Cognitive Bias Modification: Results of a Randomized Controlled Trial in an Inpatient Setting. Psychother. Psychosom..

[56] McMillan K.A., Asmundson G.J. (2016). PTSD, social anxiety disorder, and trauma: An examination of the influence of trauma type on comorbidity using a nationally representative sample. Psychiatry Res..

[57] Collimore K.C., Carleton R.N., Hofmann S.G., Asmundson G.J. (2010). Posttraumatic stress and social anxiety: The interaction of traumatic events and interpersonal fears. Depress. Anxiety.

[58] Weathers F.W., Bovin M.J., Lee D.J., Sloan D.M., Schnurr P.P., Kaloupek D.G., Marx B.P. (2018). The Clinician-Administered PTSD Scale for DSM–5 (CAPS-5): Development and initial psychometric evaluation in military veterans. Psychol. Assess..

